# The need of innovation and of preservation of well-established techniques in the era of MDR for improving outcomes

**DOI:** 10.1530/EOR-2026-0055

**Published:** 2026-05-01

**Authors:** Max Gordon, Olof Wolf, Viktor Mili-Schmidt

**Affiliations:** ^1^ Karolinska Institutet, Department of Clinical Sciences at Danderyd Hospital, Unit of Orthopedics, Stockholm, Sweden; ^2^ Department of Surgical Sciences, Orthopaedics & Hand Surgery, Uppsala University, Uppsala, Sweden

**Keywords:** medical device regulation (MDR), orthopaedic implants, clinical evaluation, equivalence, innovation, patient safety, post-market surveillance, health policy

## Abstract

- The European Union Medical Device Regulation (MDR 2017/745) has introduced stricter requirements for clinical evidence, limited reliance on equivalence and expanded post-market surveillance for orthopaedic implants.
- While designed to improve patient safety following high-profile device failures, MDR implementation has been associated with longer certification timelines, increased costs and withdrawal of established implants, particularly in lower-volume indications.
- The central regulatory challenge is proportionality: novel devices require robust prospective evidence, whereas long-established implants with extensive clinical track records may warrant risk-based recertification pathways.
- Uniform regulatory application risks unintended consequences for intermediate- and lower-volume indications that fall between orphan-device provisions and standard MDR requirements.
- Orthopaedic societies may play a key role in developing speciality-specific evidence guidance to support regulators and notified bodies, improving clarity while maintaining patient safety and sustainable innovation.

## Introduction

Medical devices (MDs) have revolutionised orthopaedics, enabling a paradigm shift from external immobilisation to sophisticated surgical reconstructions of bones and joints. Among these advancements, total hip replacement stands out as a landmark achievement, often hailed as the ‘operation of the century’, for its transformative impact on patient mobility, pain relief and quality of life (1).

However, orthopaedic innovation has also revealed important vulnerabilities. A notable example is the widespread adoption of metal-on-metal hip resurfacing, particularly in female patients, which introduced previously unrecognised failure modes and unacceptable revision rates (2, 3). These implants were frequently approved through equivalence pathways to established hip arthroplasty systems, yet simultaneously marketed as innovative solutions offering superior longevity, bone preservation and performance in younger patients (4). This disconnect, regulatory equivalence combined with claims of clinical novelty exposed patients to risks that had not been adequately evaluated through long-term clinical studies.

The need for regulation in medicine has long been recognised. Patients, lacking the expertise to distinguish genuine medical advances from persuasive claims, rely on regulatory frameworks to ensure safety and efficacy. In response, the United States introduced the first comprehensive MD regulations in 1976 (5), followed by Europe’s Medical Device Directive (MDD) in 1993 (6). In the EU, the Conformite Europeenne (CE) marking via the MDD would give access to the entire European Economic Area (EEA).

However, because MDs are used by licensed professionals and many were already on the market, they were historically subject to less stringent oversight than pharmaceuticals. This regulatory gap became increasingly evident in the early 21st century, as high-profile device failures, including hip resurfacing and proximal femoral locking plates, highlighted the limitations of existing systems and the need for reform.

The medical device regulation (MDR) (7) was introduced to address these shortcomings, replacing the MDD with stricter requirements for clinical evidence, post-market surveillance and transparency. Initially planned for full implementation in 2020, the MDR transition has faced multiple delays and is now expected to be fully enforced by 2028 (8). During this period, manufacturers have encountered substantial challenges in adapting to the new regulatory framework, exposing tensions between the promotion of innovation and the preservation of well-established, evidence-based orthopaedic practices.

This review critically examines the challenges posed by the MDR in orthopaedics, with a particular focus on balancing meaningful innovation with patient safety and long-term clinical evidence. By analysing historical precedents, regulatory evolution and early implementation experience, we aim to provide insights into how the orthopaedic community can navigate these tensions to improve outcomes and maintain trust in MD innovation.

### Regulatory architecture under MDR

Under Regulation (EU) 2017/745, MDs are classified according to risk from Class I (low risk) to Class III (highest risk). Class I devices are generally self-certified by the manufacturer; in orthopaedics, a typical example would be a non-invasive orthosis, such as a walker boot. Most orthopaedic implants, however, fall within Class IIb or III, with joint replacements and spinal implants explicitly classified as Class III (7 Annex VIII, Rule 8). Certain long-established fixation devices, such as Kirschner wires, are specifically excluded from this highest classification, reflecting a degree of proportionality within the risk-based framework.

For Class II and III devices, manufacturers must undergo conformity assessment by an independent notified body, which audits the quality management system and reviews the clinical evaluation before issuing CE certification. Clinical evidence requirements are strengthened under Article 61 and Annex XIV (7). All devices must undergo clinical evaluation to demonstrate conformity with safety and performance requirements. For implantable and Class III devices, clinical investigations are generally expected unless reliance on existing clinical data is adequately justified, and in practice, this has raised expectations for prospective evidence and longer-term follow-up compared with lower-risk classes.

All Class IIa, IIb and III devices are subject to comprehensive post-market surveillance under Articles 83–86 (7). For Class IIb and Class III devices, manufacturers must update a Periodic Safety Update Report (PSUR) at least annually, while Class IIa devices require updates at least every two years (7 Art. 86). These obligations apply irrespective of how long a device has been on the market.

Reliance on equivalence has been substantially restricted. Manufacturers must demonstrate clinical, technical and biological equivalence, together with access to the technical documentation of the comparator (7 Art. 61(4), Annex XIV §3). In practice, this limits equivalence largely to devices from the same manufacturer, particularly in orthopaedics where even minor changes in geometry, materials or fixation principles may preclude it.

These structural elements represent a substantial evolution from the previous regulatory framework under the MDD.

### What changed from MDD

The Medical Device Directive (MDD 93/42/EEC) was a directive requiring transposition into national law by each member state (6). In contrast, the MDR is directly applicable across the EU without national adaptation, resulting in greater legal harmonisation.

A major structural reform concerns the governance and designation of notified bodies. Under the MDD, manufacturers could select among numerous notified bodies with varying levels of scrutiny, contributing to inconsistencies in conformity assessment. Following high-profile device failures (9), oversight of notified bodies was substantially strengthened. Under MDR, notified bodies are jointly designated and supervised by national authorities and the European Commission and must meet stricter competence requirements. This has led to a significant reduction in their number and increased scrutiny during conformity assessment.

Another important change is the tightening of the equivalence pathway. Under the MDD, reliance on equivalence was more permissive. Concerns about such pathways are not unique to Europe; in the United States, a large proportion of recalled devices had been cleared through the 510(k) process (10). Empirical analyses have demonstrated that implants appearing superficially identical may differ in clinically relevant ways. For example, a detailed assessment of generic femoral stems modelled on the widely used Exeter design revealed measurable differences despite near-identical external appearance (11).

In addition, certain device categories have been explicitly up-classified. Joint replacements and implants in contact with the spinal column are now clearly designated as Class III under Annex VIII, Rule 8. Furthermore, Rule 11 introduced a specific classification framework for medical software, placing most software that influences diagnosis or treatment into at least Class IIa, thereby extending MDR requirements to statistical models and digital decision-support tools used in clinical practice (7 Annex VIII, Rule 11).

### Market and certification impact

In a longitudinal study examining the effects of the MDR, the typical time required for quality management system (QMS) certification increased from 6–12 months to 13–18 months between 2023 and 2025, with reported initial costs of approximately €80,000 and annual maintenance costs of €25,000 per implant (12). MedTech Europe estimates that manufacturers face an overall increase of approximately 50% in certification costs under MDR (13). As certifications must be renewed every five years, the cumulative financial impact is substantial. These regulatory and economic pressures have led many manufacturers to withdraw orthopaedic implants from the European market, particularly in niche areas with low volume. Over the past five years, major American manufacturers, such as DePuy, Stryker and Integra, have withdrawn their thumb, wrist and radial head prostheses from the European market. This decision reflects both the limited financial viability of the European market and the increased regulatory complexity introduced by the MDR (14). Launch strategies have also shifted. Larger manufacturers have reduced the EU as their primary launch geography by nearly 50% (13), with the United States increasingly becoming the first market of entry. Smaller manufacturers appear less likely to shift launch geography, potentially reflecting limited access to alternative regulatory pathways rather than strategic preference.

A further important observation is that approximately half of the total certification effort is reportedly spent on preparation for conformity assessment rather than on the assessment itself (13, p. 59). Qualitative research has highlighted substantial uncertainty among manufacturers regarding evidentiary expectations and variability between notified bodies, particularly in relation to clinical evaluation requirements (15). Within a framework of heightened accountability and joint audit, such interpretive ambiguity may contribute to increased documentation effort and longer review timelines.

Taken together, these developments suggest that MDR has altered the economic and strategic landscape for orthopaedic device certification within the EU.

### Innovation

Concerns regarding withdrawal of legacy implants have begun to materialise, as manufacturers reassess the economic viability of maintaining extensive product portfolios under MDR, with some reports describing reductions of up to one-third of available devices (16). Professional organisations, including the British Orthopaedic Association, have argued that long-established ‘gold standard’ implants with extensive clinical track records may warrant proportionate evidentiary requirements (17). A related concern is that increased certification costs may shift a greater proportion of research and development resources towards regulatory compliance, potentially reducing investment capacity for new product development. These costs may ultimately be reflected in device pricing, with possible implications for healthcare system sustainability.

The introduction of ‘orphan device’ provisions for conditions affecting fewer than 12,000 individuals annually in the European Union represents an attempt to address access for rare indications (18). However, a spectrum of lower-volume orthopaedic conditions exists that may fall outside both high-volume markets and formal orphan designation. For such indications, the cumulative costs of certification, production and distribution may challenge commercial viability, raising questions regarding proportional regulatory approaches across device categories.

An important counterpoint is that stricter equivalence requirements may strengthen incentives for genuine innovation. Manufacturers introducing products with demonstrable clinical benefits may face reduced competition from incremental ‘me-too’ devices relying primarily on equivalence claims. In the United States, analyses have shown that of more than 10,000 orthopaedic devices introduced between 2000 and 2019, only 61 were original submissions (19, 20). A high proportion of incremental submissions may limit incentives for substantial design innovation, suggesting that tighter equivalence pathways could have complex effects on competitive dynamics.

At the same time, incremental devices are not inherently problematic. In mature mechanical technologies with well-characterised biomechanics and long clinical track records – such as conventional plate fixation systems or sliding hip screw constructs – additional manufacturers may contribute to price competition, supply resilience and minor iterative refinements. The regulatory challenge therefore lies not in eliminating incremental competition but in distinguishing between low-novelty replication of established mechanical principles and genuinely novel design, material or biological innovations associated with greater uncertainty.

### Ethical implications of outsourcing clinical evaluations

The observed shift in primary launch strategies away from the European Union, attributed in part to increased regulatory complexity, cost and timelines under MDR, has implications that extend beyond market competitiveness. As manufacturers increasingly generate early clinical evidence outside the EU, this shift raises new ethical and methodological considerations.

The globalisation of clinical research is well documented, with a growing proportion of trials conducted in emerging markets, often motivated by faster recruitment and lower operational costs (21). While international research collaboration is common and frequently appropriate, variability in regulatory oversight, ethical review capacity and enforcement mechanisms across jurisdictions may influence how evidence is generated and monitored.

Differences in patient demographics, comorbidity patterns and healthcare infrastructure may also affect the external validity of clinical findings. Evidence generated in one population may not be directly transferable to another without careful assessment of contextual factors (22). When devices are first evaluated in settings substantially different from European clinical practice, additional scrutiny of generalisability may therefore be warranted.

Geographical disparities in research integrity and retraction patterns have also been documented, suggesting variability in research governance systems and publication practices across countries (23). While retraction data cannot be equated with trial quality, such findings underscore the importance of transparent reporting standards and robust methodological safeguards in globally distributed research.

Moreover, broader scholarship has highlighted how structural limitations in data access and evidentiary transparency can shape how health risks are understood and communicated (24). In the context of MDs, careful attention to the provenance, transparency and reproducibility of clinical data remains essential, particularly when regulatory frameworks rely increasingly on post-market evidence.

Taken together, these considerations do not imply deficiencies in any specific region, nor do they challenge the legitimacy of global research collaboration. Rather, they highlight the need for clear evidentiary standards, methodological transparency and regulatory cooperation to ensure that shifting launch geographies do not introduce unintended ethical or methodological vulnerabilities.

## Discussion

The historical experience of 19th-century ‘snake oil’ illustrates why regulation is indispensable in medicine. In the absence of enforceable evidentiary standards, patients are exposed to ineffective or harmful interventions. Modern device regulation represents a recalibration of the imbalance between innovation, marketing and scientific accountability. Few would argue for a return to more permissive equivalence pathways or minimal oversight. The failures associated with metal-on-metal articulations and other high-profile devices underscore that technological enthusiasm without proportionate evidence can have durable consequences for patients and healthcare systems alike. Regulation is a precondition for sustainable, trustworthy innovation.

However, the pendulum of regulatory correction may itself generate unintended distortions. Uniform recertification cycles and escalating documentation requirements for long-established implants with decades of stable performance risk conflating technological novelty with administrative renewal. When regulatory effort is expended equivalently on low-uncertainty legacy devices and high-uncertainty novel technologies, scarce evaluative capacity may be diffused rather than concentrated where risk is greatest. The emerging contraction of product portfolios and the shift of primary launch strategies outside the European Union suggest that compliance costs and procedural unpredictability are influencing strategic behaviour. In such a context, the objective should not be deregulation, but proportional recalibration: risk-based recertification pathways for legacy implants; targeted allocation of scrutiny towards genuinely novel materials, articulations or biological interfaces; and greater harmonisation and predictability across notified bodies to reduce transaction costs without diluting safety standards.

A constructive path forward lies in strengthening evidentiary quality while reducing unnecessary procedural friction. Digital infrastructures enabling automated registry linkage, electronic PROM capture and pragmatic large-scale study designs could lower the marginal cost of robust clinical evidence generation. Structured, speciality-specific evidence frameworks developed collaboratively between regulators and orthopaedic societies may improve predictability and align expectations with technological uncertainty and patient risk. Targeted structural support for SMEs and proportionate pathways for low- and intermediate-volume indications would help preserve access in niche clinical areas without undermining safety. Finally, incentives that recognise substantial investment in prospective clinical studies could discourage superficial duplication strategies while protecting meaningful innovation. In this way, the regulatory system can avoid both historical permissiveness and contemporary overcorrection, sustaining a balanced ecosystem in which patient protection, scientific integrity and responsible orthopaedic innovation remain mutually reinforcing.

### Key recommendations

(i) Structured speciality-specific evidence frameworks

European orthopaedic societies could contribute transparently developed consensus frameworks outlining clinical evidence considerations across major implant categories, such as fracture fixation systems, primary joint replacements, revision or tumour prostheses, and devices incorporating novel materials or biological interfaces. Developed in collaboration with regulators, such guidance may improve predictability in conformity assessment and support proportional calibration of evidentiary expectations to technological uncertainty, indication complexity and patient risk.

(ii) Risk-based recertification pathways for legacy devices

Implants with decades of stable clinical performance and extensive longitudinal data could be subject to differentiated recertification processes that emphasise cumulative evidence and targeted surveillance rather than uniform full reassessment cycles. Such an approach could reduce duplication of documentation and free regulatory capacity for evaluation of higher-risk innovations.

(iii) Refinement of pathways for low-volume indications

Beyond formal orphan device provisions, consideration may be given to proportionate approaches for intermediate-volume indications where clinical need persists but commercial viability is limited.

(iv) Incentives for high-quality evidence generation

Mechanisms that recognise and protect substantial investment in prospective clinical studies may help preserve incentives for meaningful innovation and discourage purely incremental duplication strategies.

(v) Development of efficient digital evidence infrastructures

Modernised approaches to evidence generation, including electronic PROM capture, automated linkage to outcome databases and pragmatic large-scale study designs, could substantially reduce the cost of producing robust clinical data while maintaining methodological standards.

(vi) Targeted structural support for SMEs

Standardised documentation templates, shared methodological resources and advisory platforms may help smaller manufacturers navigate compliance requirements without disproportionate administrative burden.

### Fact box snake oil

During the 19th century, medical markets in Europe and the United States operated with minimal statutory oversight. In this environment, patent medicines were aggressively marketed to the public, frequently accompanied by expansive therapeutic claims unsupported by evidence. These products, today often referred to as ‘snake oil’, were sold directly to consumers, with limited ingredient transparency and no requirement for premarket demonstration of safety or efficacy. Although the products were seldom innovative, their commercialisation strategies were sophisticated. Vendors marketed mixtures of opiates, cathartics and alcohol as universal remedies. These preparations consisted predominantly of psychoactive or purgative substances capable of producing symptomatic effects, which could be misrepresented as a therapeutic benefit. The commercial success of these products cannot be attributed solely to consumer credulity. Rather, it reflected structural deficiencies in regulatory governance. In the absence of enforceable standards, a marketplace emerged in which promotional rhetoric routinely outpaced scientific validation. Mounting public health concerns, investigative journalism and professional advocacy ultimately catalysed legislative reform, leading to the MDD and MDR in Europe. Modern pharmaceutical and medical device regulation can be interpreted as a response designed to recalibrate the balance between innovation, marketing and scientific accountability. Nevertheless, regulatory systems can impose evidentiary and administrative burdens that risk constraining innovation.

## ICMJE Statement of Interest

The authors declare that there is no conflict of interest that could be perceived as prejudicing the impartiality of the work reported.

## Funding Statement

This work did not receive any specific grant from any funding agency in the public, commercial or not-for-profit sector.
